# Congenital Vascular Malformations in Children: From Historical Perspective to a Multidisciplinary Approach in the Modern Era—A Comprehensive Review

**DOI:** 10.3390/children11050567

**Published:** 2024-05-08

**Authors:** Frédérique C. M. Bouwman, Bas H. Verhoeven, Willemijn M. Klein, Leo J. Schultze Kool, Ivo de Blaauw

**Affiliations:** 1 Department of Pediatric Surgery, Amalia Children’s Hospital, Radboud University Medical Center, 6525 GA Nijmegen, The Netherlands; frederique.bouwman@radboudmc.nl (F.C.M.B.); bas.verhoeven@radboudumc.nl (B.H.V.); 2 Department of Medical Imaging, Radboud University Medical Center, 6525 GA Nijmegen, The Netherlands; willemijn.klein@radboudumc.nl (W.M.K.); leo.schultzekool@radboudumc.nl (L.J.S.K.); 3 Hecovan Center of Expertise for Hemangiomas and Vascular Malformations Nijmegen, VASCERN VASCA European Reference Center, Amalia Children’s Hospital, Radboud University Medical Center, 6500 HB Nijmegen, The Netherlands

**Keywords:** congenital vascular malformations, arteriovenous malformations, venous malformations, lymphatic malformations, sclerotherapy, pediatric surgery, sirolimus, health-related quality of life

## Abstract

Congenital vascular malformations (CVMs) are the result of an aberrant development during embryogenesis. Although these lesions are present at birth, they are not always visible yet. Once symptomatic, patients suffer from pain, bleeding, ulcers, infections or lymphatic leakage, depending on the subtype of vessels involved. Treatment includes conservative management, surgery, sclerotherapy, embolization and pharmacological therapy. The clinical presentation varies widely and treatment can be challenging due to the rarity of the disease and potential difficulties of treatment. This review gives an overview of the historical developments in diagnosis and classification and exposes the key elements of innovations in the past decades on the identification of genetic mutations and personalized treatment. These advances in the field and a multidisciplinary approach are highly valuable in the optimization of clinical care aimed at both curing or stabilizing the CVM and pursuing physical and psychosocial wellbeing.

## 1. Introduction

Congenital vascular malformations (CVMs) are rare developmental anomalies of the vascular system with an overall estimated prevalence of 1–1.5% [1,2,3]. These malformations may manifest at birth or become apparent later in life as the body matures. The clinical presentation varies widely and treatment can be challenging. This review explores the complex nature of CVMs and delves into the evolving landscape of diagnostic and therapeutic advancements. Moreover, it evaluates reports on health-related quality of life, considering both the physical and psychosocial impact on pediatric patients. This overview demonstrates the necessity for multidisciplinary expertise and a holistic approach to patient care.

## 2. History

The earliest accounts of vascular anomalies can be traced back to ancient Greek history. The Hippocratic corpus already contained descriptions of the lymphatic system, leg ulcerations in venous disease and birthmarks in newborns, with the explanation that these birthmarks occurred due to maternal cravings or imaginations during pregnancy [4]. It took until the 19th century before vascular anomalies were further described and explored anatomically. The Scottish surgeon John Bell (1763–1820) described an arteriovenous malformation with the term “aneurysm by anastomosis” in his book “Principles of Surgery” [4]. The French dermatologist Jean-Louis-Marc Alibert (1768–1837) introduced a classification method for cutaneous lesions that is similar to what is used in botany, with a tree of families, genera, and species. He used the family term “angioses” for a wide variety of cutaneous lesions originating from the vascular system [4]. Rudolf Virchow (1821–1902), the founder of cellular pathology, called all vascular anomalies “angiomas” and categorized them based on the channel architecture: angioma simplex (composed of increased capillaries), angioma cavernosum (normal vasculature with large channels), or angioma racemosum (composed of markedly dilated and interconnected vessels). He envisioned that these lesions could transform into another subtype. Wegner, a student of Virchow, proposed a similar categorization for lymphatic anomalies: lymphangioma simplex, lymphangioma cavernosum, and lymphangioma cystoides. Briefly, the terminology in history was confusing and a common nosologic tongue was crucial for professionals from various specialties to collaborate and advance in research and clinical practice.

An important change in history is the foundation of the International Society for the Study of Vascular Anomalies (ISSVA) in 1976. Among the primary aims of the ISSVA was the development and continuous revision of the classification of vascular anomalies. Research focused on a combination of clinical presentation, histopathological findings and cellular kinetics, and it was discovered that there are two major types of vascular anomalies: vascular tumors and vascular malformations. Vascular tumors, among which are infantile hemangiomas, demonstrate endothelial hyperplasia. Vascular malformations are characterized by improper development of the vasculature with a normal endothelial turnover. Moreover, vascular tumors and malformations are distinct entities regarding treatment options and prognosis. Based on these findings, Mulliken and Glowacki proposed a classification in 1982, in which vascular malformations were separated from infantile hemangiomas and other vascular tumors [5]. This classification has been updated by ISSVA ever since [6].

## 3. Etiology

Congenital vascular malformations are the result of an aberrant development during embryogenesis [7]. Vascular development consists of two consecutive phases: during vasculogenesis, the blood vessels grow from embryonic cells, and during angiogenesis, the primitive vascular system extends and matures into arteries and veins [8]. In the case of congenital vascular malformations, there is an arrest or defect affecting the vascular development in early angiogenesis, between four and ten weeks of gestation [9]. This is caused by a genetic mutation, either somatic (in most cases) or germline, that directly alters intracellular signaling.

The majority of mutations identified in vascular malformations occur within two key intracellular signaling pathways: the RAS-MAPK and PI3K/AKT/mTOR pathways [10]. The mutations lead to upregulated signaling and consequently enhanced (lymph)angiogenesis. The latest ISSVA classification also includes the known causal genes of vascular anomalies. For example, MAP2K1 mutations (RAS-MAPK pathway) are associated with extracranial arteriovenous malformations and fistulas. Mutations in the RASA1 gene (RAS-MAPK pathway) are associated with combined capillary-arteriovenous malformations and Parkes-Weber syndrome, which is characterized by a combination of limb overgrowth with capillary and arteriovenous malformations. Mutations in TIE2/TEK (PI3K/AKT/mTOR pathway) have been identified in venous malformations and blue rubber bleb nevus syndrome. PIK3CA gene mutations (PI3K/AKT/mTOR pathway) are associated with lymphatic malformations, venous malformations, and PIK3CA-related overgrowth syndromes, such as CLOVES (congenital lipomatous overgrowth, vascular malformations, epidermal nevi, and skeletal/spinal abnormalities) and Klippel-Trenaunay (a combination of capillary malformation, venous and/or lymphatic malformation, and disturbed growth of bone or soft tissue).

## 4. Subtypes of Congenital Vascular Malformations

Congenital vascular malformations (CVMs) encompass a wide range of lesions and these are further categorized based on hemodynamic characteristics and the type of vessels included: arteries and veins (arteriovenous malformations or fistulas; AVMs), veins (venous malformations; VMs), lymphatic vessels (lymphatic malformations; LMs), capillaries (capillary malformations) or a combination of these, for example veins and lymphatic vessels (venolymphatic malformations; VLMs) [11]. AVMs are high-flow lesions due to the arterial component; the other abovementioned malformations are low-flow lesions. In rare cases, CVMs manifest as part of a syndrome with distinctive features.

CVMs are present at birth, although they are not always visible yet. They can manifest in various tissues, often affecting the skin and soft tissue, and occasionally bones and viscera [11]. The appearance of the vascular malformation is subject to the anatomical location, the type of vessels involved, and the timing of the arrest in development. The clinical course of CVMs is dynamic and unpredictable, although the lesions have a tendency to grow steadily as the body matures and do not show spontaneous involution [12]. In addition, sudden expansion may be triggered by infection, hormonal changes, or trauma [13].

### 4.1. Arteriovenous Malformations

Arteriovenous malformations are the most complex congenital vascular malformations and account for 3–20% of all CVMs [14,15]. AVMs are characterized by a nidus, a direct communication between dysplastic and immature arteries and veins without a capillary bed in between. As a result, the high-velocity blood flow from the arterial vasculature directly drains into the low-resistance venous vasculature. AVMs appear as a red or bluish colored soft tissue swelling with a palpable thrill. Patients suffer from pain, bleeding and ulcers due to venous hypertension or due to the principle of steal. Eventually, patients with longstanding high volume arteriovenous shunting may suffer from cardiac failure.

**Figure 1:**
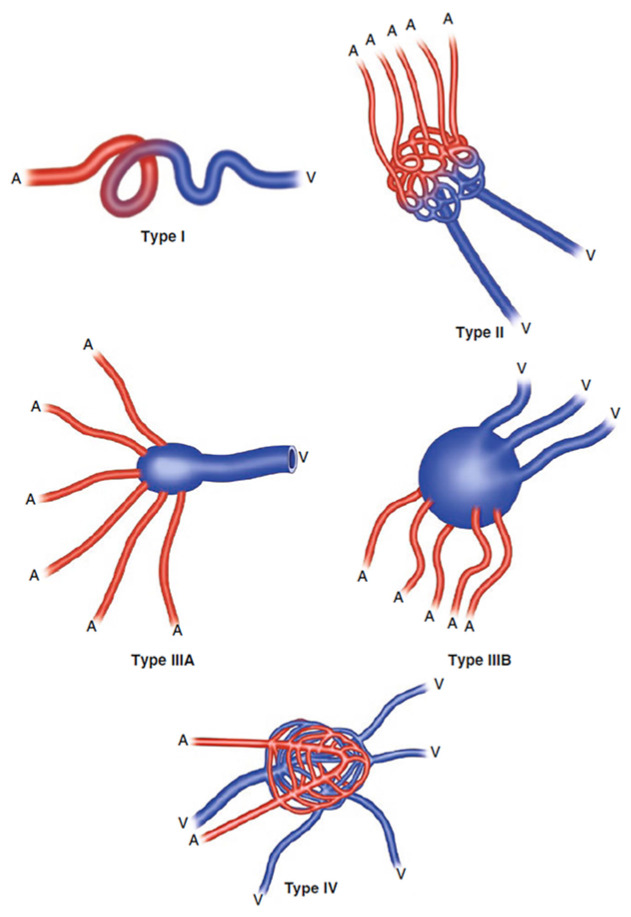
Classification of AVM angioarchitecture as proposed by Yakes et al. [17]; A = arterial vasculature (red), V = venous vasculature (blue); Type I: direct arteriovenous fistula. Type II: multiple inflow arteries into a nidus pattern with direct artery/arteriolar to vein/venular structures that may, or may not, be aneurysmal. Type IIIa: multiple arteries/arterioles into an enlarged aneurysmal vein with an enlarged outflow vein. Type IIIb: multiple arteries/arterioles into an enlarged aneurysmal vein with multiple dilated outflow veins. Type IV: innumerable micro-AV connections infiltrating an entire tissue.

Schöbinger et al. developed a clinical staging system for AVMs, based on the severity of symptomatology and the natural progression (Table 1) [12,16]. The angioarchitecture of the AVM can also be classified, based on the architecture of the nidus with the number of feeding arteries and draining veins (Figure 1) [17]. These classifications provide clinical guidance to determine the appropriate timing and approach for AVM treatment.

### 4.2. Venous Malformations

Venous malformations account for the majority of vascular malformations [14,15]. VMs appear as compressible soft tissue swelling, bluish colored in the case of superficial lesions. They expand in the dependent position or in response to factors that increase blood flow or venous pressure within the lesion, such as temperature. VMs consist of serpentine venous channels with variable luminal diameters, although often ectatic. Moreover, valves are absent or insufficient, which results in the stasis of blood flow. This may lead to phleboliths, which is pathognomonic for VMs and present in almost 50% of the lesions [18,19,20]. The main symptom of VMs is pain due to swelling and intralesional thrombosis. Extensive VMs are associated with localized intravascular coagulation, expressed as increased systemic levels of D-dimers and reduced fibrinogen levels [21].

VMs can be categorized based on the characteristics of venous drainage and connection with the normal vasculature. In 2003, Puig et al. suggested the following phlebographic classification for VMs: type I was defined as an isolated VM without peripheral drainage, type II as a VM that drains into normal veins, type III as a VM that drains into dysplastic veins, and type IV as a VM with diffuse dysplastic venous ectasia [22].

Although most VMs fall under the category of common VMs within the ISSVA classification, in some cases VMs appear as components of syndromes. For example, Klippel-Trenaunay syndrome is characterized by a large capillary malformation in combination with VMs and LMs and limb hypertrophy. Blue rubber bleb nevus syndrome is another rare congenital condition, characterized by multiple small VMs affecting the skin, soft tissues, and gastrointestinal tract [11]. In addition, a rare form of VM, known as fibro-adipose vascular anomaly (FAVA), manifests intramuscularly. It is distinguished from a common intramuscular VM by features of fibrofatty accumulation, diffuse fibrotic infiltration of the muscle, extension along fascial planes and consequently contracture of the extremity.

### 4.3. Lymphatic Malformations

Lymphatic malformations account for 10–30% of all CVMs [1,14,15]. Most LMs are identified at birth or usually within the first two years of life and appear as a soft, compressible lesion. Symptoms include pain, swelling, intralesional bleeding, lymph leakage, and relapsing periods of infection. Lymphatic malformations can be subclassified into macrocystic, microcystic, or mixed lesions. The difference between macrocystic and microcystic is defined by the size and accessibility of the cysts [7,23,24]. Macrocystic lesions contain cysts that are ≥2 cm or considered accessible for aspiration based on ultrasound. Microcystic lesions contain cysts that are <2 cm or considered inaccessible for aspiration. Mixed lesions contain both macrocystic and microcystic components.

**Figure 2:**
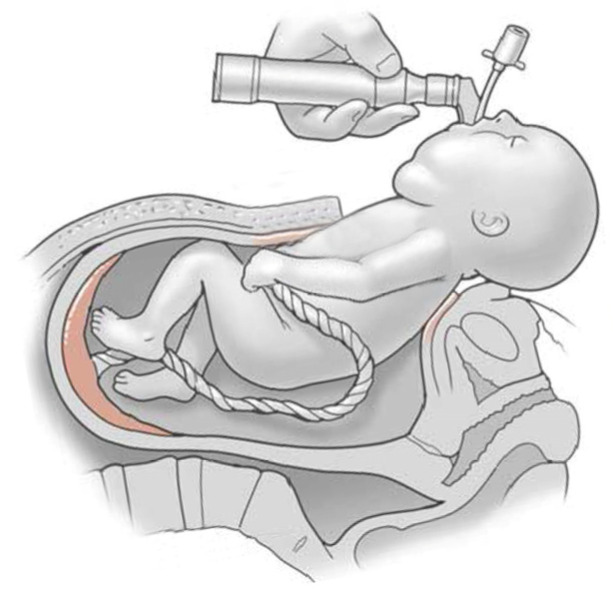
Ex-utero intrapartum treatment.

LMs are most frequently located in the head and neck area (48–75%) [25,26,27]. In rare cases, the LM is located adjacent to the airway and already detected prenatally. In case an endangered airway is suspected immediately after birth due to the LM, ex-utero intrapartum therapy (EXIT) may be performed to secure the neonatal airway under placental support (Figure 2) [28].

As in VMs, the majority of LMs can be classified as common LMs within the ISSVA classification; however, sometimes LMs appear as complex lymphatic anomalies. For example, generalized lymphangiomatosis is characterized by a diffuse LM affecting one or multiple organ systems, and Gorham-Stout disease features a distinct pattern of an intraosseous LM with progressive osteolysis.

## 5. Diagnosis

The diagnostic workup starts with a clinical history and physical examination. Based on this information, CVMs can often be differentiated from vascular tumors, such as infantile hemangiomas or other pathologies that may mimic CVMs. Moreover, this initial assessment guides the identification of the CVM subtype it concerns. Ultrasound imaging is the next step in the diagnostic workup. It provides information about the anatomical extent and can differentiate between high-flow (AVM) and low-flow (VM and LM) vascular malformations. Furthermore, ultrasound is often used for percutaneous treatment and hence this diagnostic imaging technique gives important information about the accessibility of the lesion. In case invasive management is indicated, additional imaging could be conducted, with the choice of imaging method tailored to the specific CVM subtype. For low-flow vascular malformations, such as VM and LM, additional magnetic resonance imaging (MRI) is most appropriate. MRI gives important additional information regarding tissue differentiation, which aids in differentiating between benign and malignant tissue and between a venous and lymphatic malformation. In addition, MRI is very helpful in the assessment of the extent of the CVM and its relation with adjacent structures [29]. For AVMs, digital subtraction angiography (DSA) is the gold standard diagnostic method, owing to its capacity to confirm AV-shunting, assess the angioarchitecture of the nidus, and evaluate the specific drainage pattern. Besides its diagnostic prowess, DSA plays a pivotal role in guiding sclerotherapy and embolization. An alternative imaging method for AVMs is four-dimensional computed tomography angiography (4D-CTA). This technique combines the features of CT and angiography; with time as a fourth dimension, a dynamic image is created, which enables evaluation of the hemodynamic characteristics of the AVM [30]. However, the utility of 4D-CTA is tempered by its use of ionizing radiation, a factor that necessitates careful consideration, particularly in children.

Genetic analysis on histopathological material is indicated when the diagnosis is unclear—despite the integration of clinical history, physical examination and radiological imaging—or when the patient does not respond sufficiently to the initiated therapy. Increasingly, genetic sequencing is also used for extensive abnormalities where treatment through sclerotherapy means that frequent procedures (often under general anesthesia) are needed over a longer period of time, and pharmacological therapy could therefore be a better alternative. A genetically determined DNA mutation helps in selecting the appropriate pharmacological therapy and predicting the likelihood of symptom reduction in advance. This information also enhances counseling and shared decision making.

## 6. Treatment

### 6.1. Conservative

A conservative approach is appropriate in case of asymptomatic vascular malformations, in case of mild symptoms and/or if the risks of invasive treatment outweigh the risks of the disease [31,32]. Cosmetic camouflage may be used to improve the appearance [33]. Compression garments can reduce symptoms and possibly prevent lesion-related complications [34]. Conservative approaches also include proper skin, wound and ulcer care.

### 6.2. Surgery

In case of symptomatic lesions, invasive management could be performed to treat the vascular malformation. The traditional invasive management of vascular malformations was surgical resection. This can be challenging due to the numerous vascular connections, especially in AVMs, and infiltration of the surrounding tissue. Resection of CVMs has been associated with significant blood loss, collateral damage to the surrounding tissues (soft tissue, nerves, musculoskeletal system), wound problems, and recurrence, particularly in the case of suboptimal irradical resections [35,36,37]. Recent studies on surgical resection of CVMs mainly include VMs and LMs [38,39,40,41]. A complete response was reported in 37–94% and recurrence in 12–38%. Complication rates ranged from 9–18% and most frequently included hematoma, seroma, infection, and nerve complications. Although surgical resection is still performed nowadays [36,37,40,42,43,44], endovascular treatment is usually the primary treatment of choice. Endovascular treatment can be combined with surgery, either to perform debulking in the case of functional impairment whether or not preceded by sclerotherapy, or as the last step to remove the residual mass [45,46].

### 6.3. Sclerotherapy/Embolization

As the focus shifted towards minimally invasive treatment options, sclerotherapy and embolization emerged as alternatives to surgical resection. The technique consists of injecting chemical agents into the CVM with the aim of thrombosing and obliterating the vessels and thereby inactivating the malformation. Initially reserved for patients deemed inoperable, sclerotherapy and embolization have emerged as the treatment of choice in many cases, offering advantages over surgical resection [47,48,49,50]. Various chemicals can be used, such as ethanol, coils, glues, Onyx (ethylene-vinyl alcohol copolymer), bleomycin, polidocanol (aethoxysclerol), doxycycline, and many more [49,51,52,53,54,55,56,57,58]. The specific properties and working mechanisms of these agents differ, and the choice of which agent to use can be based on CVM subtype, anatomical location, content and extent of the lesion, or simply expertise in a center. The difference between sclerotherapy and embolization lies in the working mechanisms of the agents; embolizing agents occlude the vessels, often by mechanical means, and sclerosing agents destroy the vascular endothelium causing inflammation, clotting and fibrosis.

**Figure 3:**
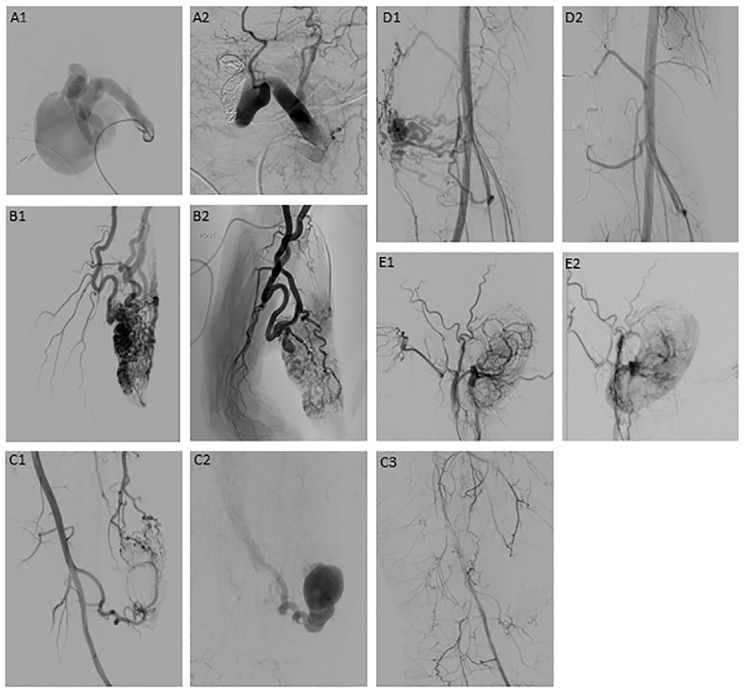
Examples of cases of AVM embolization [54]; (**A1**,**A2**) AVM type I in the face ((**A1**) before embolization, (**A2**) total occlusion after embolization); (**B1**,**B2**) AVM type II in the thumb ((**B1**) before embolization, (**B2**) partial occlusion after embolization); (**C1**–**C3**) AVM type IIIa in the lower extremity ((**C1**) before embolization, (**C2**) venous outflow before embolization, (**C3**) total occlusion after embolization); (**D1**,**D2**) AVM type IIIb in the knee (**D1**) before embolization, (**D2**) total occlusion after embolization); (**E1**,**E2**) AVM type IV in the ear (**E1**) before embolization, (**E2**) near-total occlusion after embolization).

Ethanol is a particularly powerful agent due to its protein denaturing properties, and is thereby the only agent that results in complete and permanent vessel occlusion. Figure 3 shows examples of ethanol embolization of AVMs, illustrating various angioarchitectures and partial to total devascularization of the nidus [54]. Although ethanol is the most powerful agent, some are reluctant to use ethanol because of previously described possible complications [59,60,61]. The major pitfall in utilizing ethanol is leakage into the normal vasculature, which—when prevented—reduces the risk of post-procedural tissue necrosis and increases treatment efficacy [62,63]. Polidocanol (aethoxysclerol) is an agent that can be used either as a liquid or as a foam, which has advantages in large lesions, since the efficacy of sclerotherapy depends on the quality of contact of the agent with the endothelial lining and this can be increased by using a foamed agent. Bleomycin is currently the agent of choice for LMs. It is an antibiotic derivative that exhibits cytostatic properties by inducing single- and double-strand breaks in DNA, thereby inhibiting cell division, growth and DNA synthesis. Although the exact working mechanism is unknown, it also results in endothelial damage and fibrosis. Bleomycin is safe and effective and results in less swelling compared to other agents, which is a major advantage. In some cases, it can be beneficial to use a combination of agents based on their specific properties [49,52,57].

Although sclerotherapy and embolization outweigh most of the disadvantages of open surgery, this therapy also has its drawbacks. Possible complications include wound problems, nerve injury and deep venous thrombosis. In addition, some severe systemic complications have been reported, mostly after the use of high dosages, such as pulmonary hypertension and cardiac arrest in the case of ethanol and pulmonary fibrosis in the case of bleomycin [64,65,66,67,68]. Hence, the dosage and volume of the agent utilized per session and over the patient’s lifetime are restricted. Consequently, patients frequently require multiple sessions of sclerotherapy or embolization.

Studies on treatment outcomes of sclerotherapy/embolization often focus on a specific CVM subtype, a specific anatomical location, or a specific agent, and the definition of a successful treatment outcome varies, all of which limits comparability. In recent studies on pediatric patients, a complete response was reported in 11–89% of the patients and improvement in 57–100% [34,48,49,51,54,69,70]. In LMs, bleomycin seems to be the most effective agent, and macrocystic lesions often showed a better response than microcystic and/or mixed lesions [49,53,70]. In AVMs, the success rate of embolization was influenced by the angioarchitecture of the nidus [54]. Complication rates varied from 6–25% on the procedural level and from 10–54% on the patient level in recent studies [34,48,49,51,54,69,70], with historical data reporting complication rates up to 82% on the procedural level and 72% on the patient level [50,59,71]. Complications frequently reported include skin necrosis, infections, and (mostly temporary) nerve complications.

### 6.4. Medical Therapy

In the context of CVMs, medical therapy can serve as either a supportive or complementary component to the aforementioned management options, or a primary treatment modality in contemporary practice. In a supportive or complementary role, medical therapy can be used for general consequences, such as pain, anemia, and infection, as well as addressing hemostatic complications. In patients with infections, long-term antibiotic prophylaxis can be considered [72]. Patients with localized intravascular coagulopathy have been found to benefit from subcutaneous low-molecular weight heparin or oral anticoagulants [73,74].

Over the past decade, research has increasingly focused on cellular proliferation as a therapeutic target for medical treatment, either as supportive or single therapy. For example, thalidomide is a drug with antiangiogenic effects by suppression of endothelial growth factors and it has been proved effective in refractory bleeding from gastrointestinal vascular malformations [75]. In addition, the detection of genetic mutations in vascular malformations opened up a new field of translational research with cellular proliferation as a target. For example, sirolimus is an mTOR inhibitor and several studies showed that pharmaceutical treatment with sirolimus could reduce the pain and size of the vascular malformation without surgery or endovascular therapy [76,77,78,79]. Recent studies on treatment outcomes of sirolimus in pediatric patients showed an improved HRQOL in around 80% of the patients [79,80]. Some studies used volume reduction on MRI as outcome measure; Ji et al. reported an objective response in 78% of the patients, defined as a ≥20% decrease in lesion volume, whereas Maruani et al. reported an overall significant reduction in LMs, but not in VMs and combined malformations [80,81]. Tian et al. combined clinical and radiological responses and reported a complete response in 4% of the patients and a partial response in 86% of the patients [82]. The majority of patients treated with sirolimus experienced one or multiple adverse events, although most were minor. The adverse events most frequently reported included mucositis, respiratory tract infections, headache, and metabolic toxicities [79,80,81,82].

Currently, alpelisib—a selective PI3Kα inhibitor—is being investigated and used in an experimental setting for patients with a PIK3CA or TEK mutation [83,84]. Another example is trametinib, which is a MEK inhibitor, and promising results have been reported in patients with complex lymphatic anomalies associated with mutations in the RAS-pathway [85]. Targeted pharmacotherapy is evidently gaining prominence and has already established a dominant position in current practice for specific cases. Furthermore, since the knowledge of gene mutations associated with CVMs continues to grow, medical therapy is expected to become more widely available and to play a dominant role in a broader range of cases in the future.

## 7. Quality of Life

Patients with a CVM are at risk of an impaired health-related quality of life (HRQOL) [86]. This could be attributable to the severity of the disease, social stigmatization, repetitive hospitalizations, and treatment-related morbidity. Nevertheless, it has only been in the last two decades that studies have been published on HRQOL as a patient-reported outcome. The importance of this has been confirmed by the Outcome Measures for Vascular Malformations (OVAMA) project, an international multicenter consensus study, which defined HRQOL as a core outcome measure for all patients with CVMs [87].

Seven studies quantitatively evaluated HRQOL in pediatric patients with arteriovenous, venous, lymphatic, and/or combined vascular malformations. Two large studies showed reasonable median PedsQL total scale scores of 84.8/100 parent-reported and 83.7–84.8/100 patient-reported [88,89]. One of these studies compared the PedsQL scores with normative means and concluded that an impaired HRQOL, defined by a PedsQL score > 1 standard deviation below the normative mean, was found in 25% of the patients [88]. Teenagers scored lower values on the physical domain and in a direct comparison between parents and their children, parents reported lower values mainly on the psychosocial domain. Two studies evaluated HRQOL at baseline and at follow-up, defined as respectively six to eight weeks and three months after the last treatment [90,91]. Lokhorst et al. used both the PedsQL questionnaire and the Children’s Dermatology Life Quality Index (CDLQI) [90]. The patient-reported PedsQL total scale score increased from 78.7/100 at baseline to 78.1/100 at follow-up and the CDLQI score improved from 6.4/30 to 6.1/30, although the authors concluded that both questionnaires were not sufficiently responsive for evaluating treatment effect. Wohlgemuth et al. used the SF-10 Health Survey and reported physical impairment at baseline with a physical summary score of 25.3, which increased to 45.9 after treatment [91]. The psychosocial summary score increased from 51.1 at baseline to 58.8 at follow-up. In both summary scores, a score of 50 corresponded to the average score of the general population. Another study used the KIDSCREEN questionnaire and measured HRQOL at least five years after sclerotherapy, which resulted in values that paralleled age-appropriate norms [92]. Currently, studies more often incorporate HRQOL assessment into their response evaluation [93]. This is also evidenced by recent research on sirolimus [81,94]. A study conducted in this expertise center showed that the PedsQL total scale score at baseline was significantly lower than in the general population with a mean patient-reported score of 66.3/100 and parent-reported score of 64.0/100 [94]. After six months of treatment with sirolimus, patients reported a mean total scale score of 76.2/100 and parents a mean of 74.9/100. Another study used the CDLQI and reported that the number of patients with a CDLQI score of 0–1—indicating no effect on HRQOL—increased after sirolimus treatment, from 16/59 children (27%) at baseline to 32/59 (54%) after one year [81].

Factors reported to be associated with an impaired HRQOL included invasive treatment, the number of sclerotherapy procedures, age at first treatment, and anatomical location, either in the head and neck region or in the lower extremity [88,89,92]. No specific CVM subtype seems to be independently associated with an impaired HRQOL [88,95,96,97], although some studies suggest worse HRQOL in complex CVMs associated with other anomalies [89,98]. From a clinical perspective, it could be hypothesized that patients with an AVM are particularly at risk of an impaired HRQOL, since AVMs are known to be more frequently associated with lesion-related complications, also in the cardiovascular system, and are generally more difficult to eradicate compared to other CVM subtypes. On the other hand, it is also imaginable that, for example, patients with an LM in the head and neck region or with excessive lymphatic fluid leakage or patients with a VM with localized intravascular coagulopathy suffer from physical or psychosocial distress. Patients with a CVM associated with other anomalies probably have a higher risk of an impaired HRQOL, although statistical significance may be limited by small subgroups.

## 8. Future Perspectives

Sclerotherapy and embolization are generally safe and effective in the majority of patients with, on average, a reasonable HRQOL. However, a subset of patients does not respond well to conventional treatment and/or suffers from an impaired HRQOL.

The advancements in the detection of genetic mutations and the subsequent development of targeted treatment have fundamentally changed clinical care over the past decade. Where embolization and sclerotherapy overcame most disadvantages of surgical resection and became the primary treatment of choice in most cases, systemic treatment with the mTOR inhibitor sirolimus has already evolved from last-resort treatment in an experimental setting to the primary treatment of choice in certain patients. Moreover, the promising results of sirolimus have led to the investigation of alpelisib, a selective PI3Kα inhibitor.

With increasing knowledge of molecular biology and the pathophysiology of CVMs, specific patient or lesion characteristics are likely to adopt a central role in guiding treatment decisions. Genetic analysis of the lesion is set to become increasingly critical, enabling the determination of the most effective treatment strategy and advancing the shift towards personalized medicine. Moreover, this could aid in the shared decision-making process, helping to decide whether a step up approach is suitable, if a specific treatment modality should be prioritized, or how various treatment options can be integrated to improve patient outcomes. Further research is essential to expand the evidence on which treatment is best and to further explore targets for personalized treatment, with the aim of optimizing effectiveness and minimizing adverse events.

Although procedure-related complication rates associated with sclerotherapy and embolization are relatively low in general, the complication risk—in particular in repetitive sessions, which is often necessary—should not be ignored. Centralization of care and a multidisciplinary approach are key in the optimization of treatment outcomes in these patients. With regard to treatment, it would be interesting to investigate how the efficiency of embolization therapy can be optimized, since the patient complication risk is obviously increased in case of repetitive embolizations, and to explore the optimal timing of treatment. Would an early intervention be favorable or would it be better to wait until the risks of general anesthesia are lower in case of a young child or when the symptoms of the disease outweigh the risks of treatment? Although it is difficult to specifically investigate this due to the clinical variability, it would be a next step in the development of tailored treatment plans. For example, initiating sirolimus treatment early could be particularly advantageous for neonates born with an LM adjacent to the airway, especially those presenting with factors indicating a likelihood of poor outcomes. Furthermore, the efficacy of sirolimus has been observed in two case reports, where sirolimus was started during pregnancy as fetal management [99,100]. In addition, it is worth considering standardizing a multidisciplinary reassessment after, for example, three sclerotherapy/embolization procedures. With the ongoing developments in diagnostics and treatment options, patients with a poor response to regular treatment might benefit from targeted treatment.

Regarding treatment outcomes, various outcome measures have been reported in the literature to describe the effectiveness and complications of CVM treatment. International consensus on which outcome measures and grading systems to use would be valuable in order to optimize comparability and generalizability. The OVAMA project (Outcome measures for VAscular MAlformations) already made a major contribution by developing a core outcome set [87]. Now it is important to apply these core outcome measures in current studies and to reach consensus about a uniform approach regarding grading systems and questionnaires. Currently, VASCA is working on an international registry making use of the FAIR data principles: findable, accessible, interoperable, and reusable. With the continuous advances in the field and the aim of optimizing care by centralization, it is crucial to have a profound insight in the current techniques and outcomes of all treatment centers, using such a systematic approach and including both short-term and long-term results.

Patient-reported outcome measures are expected to play a more prominent role in the evaluation of treatment outcomes in the future. The disease-specific OVAMA questionnaire has been validated and this questionnaire also includes patient satisfaction with the treatment strategy and outcome [101]. The complementary use of a generic and disease-specific questionnaire will allow a comparison with normative means and other diseases, while also achieving specificity. In addition, it would be interesting to explore patient-reported weighted scores, in which patients themselves rate to what extent each item contributes to their wellbeing. These questionnaires can be completed at baseline and at certain timepoints. The next step is to transform these results into a tailored clinical care pathway. Based on differences found between parental and children’s ratings of HRQOL, it is important to address both children and their parents during their visits and to take into account family impact. Furthermore, patients who suffer from an impaired HRQOL or patients who are at risk based on factors associated with impairment may benefit from psychosocial or rehabilitation support. It could be valuable to introduce a specific program for these patients, in which they are supported by a pediatric medical psychologist and a pediatric rehabilitation specialist, for example in a hybrid form with online and in-person meetings.

## 9. Conclusions

In summary, this review gives an overview of the complex nature of CVMs, challenges in treatment, health-related quality of life, and recent advancements in the field. Given the complexity of CVMs, the multitude of factors involved, and the ongoing diagnostic and therapeutic advancements, a collaborative multidisciplinary approach is essential in achieving the ultimate goal: curing or stabilizing the CVM and pursuing physical and psychosocial wellbeing.

## Figures and Tables

**Table 1:** Schöbinger classification of AVM.

Stage I—quiescence	Pink-bluish stain, warmth, arteriovenous shunting on Doppler
Stage II—expansion	Stage I plus enlargement, pulsations, thrill, bruit and tortuous/tense veins
Stage III—destruction	Stage II plus dystrophic skin changes, ulceration, bleeding, tissue necrosis. Lytic bone lesions may occur.
Stage IV—decompensation	Stage III plus congestive cardiac failure with increased cardiac output and left ventricle hypertrophy
